# Humanizing Birth in a Third-Level Hospital: Revealing the Benefits of Natural Cesarean Sections

**DOI:** 10.3390/life14030397

**Published:** 2024-03-17

**Authors:** Paula Recacha-Ponce, Pablo Baliño Remiro, Laura García-Rayo-Reolid, Violeta Dominguez-Gomez, María Pilar Suárez-Alcázar, Ana Folch-Ayora, Pablo Salas-Medina, Eladio Joaquin Collado-Boira

**Affiliations:** 1 Faculty of Health Sciences, Jaime I University, 12071 Castello de la Plana, Spain; recacha@uji.es (P.R.-P.); malcazar@uji.es (M.P.S.-A.); afolch@uji.es (A.F.-A.); psalas@uji.es (P.S.-M.); colladoe@uji.es (E.J.C.-B.); 2 Castellon General University Hospital, 12004 Castello de la Plana, Spain; laura.g_99@hotmail.com (L.G.-R.-R.); vdgomez1@hotmail.es (V.D.-G.)

**Keywords:** well-being of mothers, well-being of infants, breastfeeding, natural cesarean section, midwifery

## Abstract

Background and Aims: Efforts to humanize childbirth focus on promoting skin-to-skin contact, labor accompaniment, and breastfeeding. Despite these advancements, cesarean sections often lack a consideration of immediate mother–child contact, early breastfeeding initiation, and follow-up. This underscores the need for a ‘natural’ approach to cesarean sections, aiming to ‘humanize’ the procedure and emulate some aspects of vaginal birth. Materials and Methods: An observational longitudinal cohort study was conducted, involving pregnant women scheduled for a cesarean section. Two comparison groups were established: one undergoing conventional cesarean sections and the other receiving a humanization intervention. While in “conventional cesarean sections,” newborns are separated from mothers at birth, preventing actions such as early breastfeeding or skin-to-skin contact, and maternal companionship is lacking in the operating room, the intervention of cesarean section humanization was based on avoiding the separation of the mother and newborn, promoting skin-to-skin contact, early breastfeeding, and maternal accompaniment during surgery. Descriptive data on maternal and neonatal variables, including breastfeeding initiation, maintenance, and baby weight trends, were collected. Additionally, a validated survey assessed the pain, satisfaction, and anxiety among the 73 participating women. Results: Women undergoing natural cesarean sections reported higher satisfaction, lower anxiety, and reduced postoperative pain, requiring less analgesia. Although their exclusive breastfeeding rates at 10 days postpartum showed no significant difference, statistically significant differences favored natural cesarean sections at 3 months (67.5% vs. 25%) and 6 months (50% vs. 4.5%). Neonates in the natural cesarean group exhibited greater weight gain at 10 days postpartum compared to those delivered conventionally (+49.90 g vs. −39.52 g). No significant differences in blood counts were observed between the groups. Conclusions: This study underscores the manifold advantages offered by the natural cesarean procedure compared to the conventional cesarean approach. Notably, a NC demonstrates superior outcomes in terms of heightened maternal satisfaction with the obstetric process, the enhanced sustainability of exclusive breastfeeding, and augmented neonatal weight gain.

## 1. Introduction

With the aim of humanizing childbirth, the World Health Organization (WHO) and UNICEF launched the Baby-Friendly Hospital Initiative (BFHI), which has been updated several times [1,2,3]. The recommendations contained in these and other guidelines [4] have been implemented in the context of vaginal birth to promote the maximum humanization and naturalness of the process. In addition, these guidelines have also been considered in the context of cesarean sections [5]. Contrary to what might be expected, the number of cesarean sections has continued to increase in recent years [6,7], despite the WHO recommendation that no more than 10–15% of births should be by this method [8,9]. Measures to humanize vaginal delivery should have been implemented in line with this trend. However, despite their proven safety in the operating theatre, more ‘humanized’ or ‘natural’ approaches, such as skin-to-skin contact and an initiation of breastfeeding within 30 min [2], are rarely used in cesarean sections [10].

In 2018, the WHO published ‘Intrapartum care for a positive childbirth experience’, which provides guidelines for normal childbirth to promote maternal and fetal well-being and advance the new Global Strategy for Women’s, Children’s and Adolescents’ Health (2016–2030) [11]. The guidelines recognize that a ‘positive birth experience’ is an important goal for all women who give birth. It defines a positive birth experience as one that meets or exceeds the woman’s prior personal and sociocultural beliefs and expectations, including the birth of a healthy baby in a safe clinical and psychological environment, with ongoing practical and emotional support from birth companions and from kind and technically competent clinical staff. It is based on the premise that most women want a physiological labor and birth and a sense of personal achievement and control through their participation in decision making, even when medical interventions are needed or desired. These updated, comprehensive, and consolidated guidelines on essential intrapartum care bring together new and existing WHO recommendations that, when implemented as a package, will ensure high-quality, evidence-based care regardless of the context. Some of the guidelines state that a companion of choice is recommended for all women throughout labor and childbirth; that newborns without complications should be kept in skin-to-skin contact with their mothers for the first hour after birth to prevent hypothermia and promote breastfeeding; or that all newborns, including low-birth-weight babies who are able to breastfeed, should be breastfed as soon as possible after birth when they are clinically stable and the mother and baby are ready. It seems clear, therefore, that these guidelines should be extended to cesarean births.

The benefits of these practices have been widely demonstrated [12,13], and this approach should be extended to cesarean sections, where maternal–fetal conditions allow. This type of cesarean section is also known as “gentle caesarean” [14], “natural caesarean” [14] or “family-centred caesarean” [15].

The main features of this type of cesarean section are that the newborn is immediately placed skin-to-skin on the mother’s breast, and separation is avoided in order to encourage early breastfeeding and promote emotional bonding between the mother and child. The atmosphere created is similar to that of vaginal birth, where the couple participates in the process by taking on an active role [5,11,16]. This is contrary to what happens in “conventional cesarean sections”, where the newborn is separated from its mother at birth, does not engage in skin-to-skin contact or early breastfeeding, and, furthermore, the woman is not accompanied by the person of her choice.

The current literature shows that this type of cesarean section has benefits, such as an increase in family satisfaction after the procedure [17]. However, institutions seem reluctant to implement them in their obstetric services, so it is fair to equate vaginal delivery with a cesarean section in terms of maternal and neonatal outcomes. The rationale for this study is to demonstrate the potential benefits of a natural cesarean section and thereby promote its adoption in the obstetric services of as many hospitals as possible.

The main aim of this project is to investigate the maternal/neonatal benefits of a ‘natural cesarean section’ (NC) compared to ‘conventional cesarean section’ (CC).

Therefore, the objectives of the study are as follows:

- Determine the impact of skin-to-skin contact on the initiation and continuation of breastfeeding.
- Analyze differences between types of cesareans in terms of exclusive breastfeeding at 10 days and 3/6 months after birth.
- Compare natural and conventional cesareans in terms of weight gain with exclusive breastfeeding at 10 days postpartum.
- Compare natural and conventional cesareans in terms of maternal satisfaction.

## 2. Material and Methods

### 2.1. Participants and Data Collection

This study was conducted from January to December 2020. A total of 73 women (NC: *n* = 43; CC: *n* = 30) and 81 newborns (NC: *n* = 50; CC *n* = 31) participated in the study. All participants gave their informed consent to taking part in the study.

A working group of midwives and obstetricians was specifically trained to work with the methodology of the NC section. A convenience sample of 43 patients scheduled for cesarean section, coinciding with the shifts of this team, formed the final sample of the natural cesarean section group. Urgent or emergency cesarean sections were excluded. Inclusion criteria in both study groups were women with an indication for a planned cesarean section and their willingness to participate in the study. The exclusion criteria consisted of mothers with pathologies or treatments that could affect the initiation/maintenance of breastfeeding or neonatal adaptation to extrauterine life (oncological treatments, drug consumption, etc.), in addition to a language barrier. The scheduling of these cesarean sections was based on the protocol of the Hospital Universitario Clinic de Barcelona [18]. The Robson classification was then used to classify and compare the reasons for scheduling an elective cesarean section. The Robson classification, published in 2001, is a system used to classify cesarean sections, dividing women into 10 clinically relevant groups [19]. Participants also agreed to return 10 days after delivery to assess infant weight and breastfeeding. All women had access to breastfeeding counseling throughout the study, provided by midwives and pediatricians in primary care centers.

A questionnaire developed by the research team was used to collect demographic, obstetric, and neonatal data. A validated questionnaire was used to assess satisfaction, anxiety, and pain levels [17]. The questionnaire was administered by a maternity nurse on the third postpartum day. Incorrectly completed or incomplete questionnaires were not included in the study.

Ethical approval was obtained from the hospital ethics committee (CEIM 2/2019). The study was conducted in accordance with national and international standards and the 1995 Declaration of Helsinki. The study complied with the Organic Law on Data Protection and Guarantee of Digital Rights. All participants received written information about the aims of the study and their right to refuse to participate or withdraw at any time.

Sample Size Estimation:

A sample size estimate for the study, to ensure the robustness of our statistical analysis, was calculated using the G-power statistical power analysis program. The sample size was calculated from the number of cesarean deliveries at the Hospital General Universitario de Castellón in 2019. Out of 1288 cesarean deliveries, a sample size of 77 patients was determined with a confidence level of 95% and precision of 5%, taking into account an estimated loss rate of 10%.

### 2.2. C-Section Procedure

On the day of delivery, the NC group was accompanied to the operating room by a chosen individual who remained present throughout the procedure. During the operation, the companion sat beside the mother’s bed and both were attended to by anesthesia personnel and a midwife. Throughout the intervention, the mother was covered with a sheet from side to side. The anesthesia was spinal and the incision type was horizontal. Once the amniotic sac was removed, the sheet was lowered, and the mother and family were able to maintain visual contact with the newborn, who was placed on the mother’s chest by the midwife, who at that time was part of the operating room team in a sterile manner, for early skin-to-skin contact. After this, the midwife exited the sterile area and accompanied the newborn, as well as the mother, until the surgical procedure was completed and they reached the hospital ward. A pediatrician examined the newborn on the mother’s chest. The midwife encouraged early breastfeeding attachment. Early latch was initiated with the assistance of the midwife, and this was verified through observation. It is of importance to remark that the midwives and obstetricians were specifically trained to work with this methodology and observe a proper latching. Following the procedure, the mother and newborn remained in the delivery room, accompanied by a chosen individual and supervised by the midwife.

In the CC group, participants followed the usual hospital cesarean section procedure, meaning that the pregnant woman remained alone in the operating room during the procedure. After delivery, the newborn was taken to the observation room to be examined by a pediatrician and then handed over to the family while the mother was taken to the recovery room. Approximately two hours later, the mother was taken to the room with her baby and family members. Breastfeeding was then established.

In both types of cesarean sections, a midwife is present who will be responsible for receiving the newborn. However, in conventional cesarean sections, the midwife takes the newborn to the room with the family while waiting for the mother, whereas in natural cesarean sections, it is this figure who is responsible for placing the newborn on the mother’s chest and supervising the process, ensuring their safety in the event that the mother feels unwell during surgery.

Carrying out an NC section entails a greater need for human resources, as it requires the presence of a nurse specializing in obstetrics and gynecology to accompany this process. This nurse will be responsible for receiving the baby in a sterile manner and placing them on the maternal chest, as well as holding the baby and supervising the process throughout the surgery.

### 2.3. Variables of the Study

Maternal: the main variables collected in the questionnaire focused on information about the operating room, respect for the staff, anxiety before the operation, the quality of rest the night before the cesarean section, and pain experienced during the anesthetic procedure and during and/or after the cesarean section.

Newborn: The main variables were latching, defined as a proper attachment of the newborn to the mother’s breast to ensure optimal milk transfer, early skin-to-skin contact, an early initiation of breastfeeding, NICU admission, continuation of breastfeeding at 10 days and 3/6 months, and newborn weight gain at 10 days. A previously calibrated digital scale was used to determine neonatal weight on the day of birth and at 10 days. The same scale was used at these 2 time points.

### 2.4. Data Analysis

Normalization of the data was first measured using the Kolmogorov–Smirnov test, according to which all data had a normal distribution. Data were presented as mean ± standard error of the mean.

Student’s *t*-test was used to compare the clinical and demographic variables of the participants (mothers and neonates).

Chi-squared test was used to compare Robson’s classification between groups.

Fisher’s exact test was used to determine skin-to-skin contact and spontaneous latching.

The analysis of exclusive breastfeeding at 10 days and 3/6 months was evaluated by *t*-test comparisons.

The *t*-test was used to compare hematological and neonatal weight gain values between groups. A weight gain index (WGI) (WGI = weight at 10 days − weight at birth) was defined to compare neonatal weight gains at 10 days [20].

To compare maternal hematologic variables, a delta score (Δ) was defined as follows: Δ (fold increase) = (post level value − baseline level value)/baseline level value.

For all quantitative variables, their effect size was determined by Cohen’s d test as follows: d values < 0.5 were considered to indicate a small effect, values between 0.5 and 0.8 were considered to reflect a moderate effect, and values greater than 0.8 were considered to indicate a large effect.

Questionnaire variables were compared using the Mann–Whitney U test.

Statistical analysis was performed using the Statistical Package for the Social Sciences software (IBM SPSS Statistics for Windows, version 26.0, IBM Corp., Armonk, NY, USA).

Finally, statistical significance was set at *p* < 0.05.

## 3. Results

Of the initial study participants, only one withdrew, leaving a final sample of 73 pregnant women and 81 newborns. The final participants were distributed as follows: *n* = 44 women received an NC section (59.72%), *n* = 29 women received a CC section (40.28%), *n* = 50 newborns were delivered by NC section (61.72%), and *n* = 31 newborns were delivered by CC section (38.28%). There was one multiple gestation in the CC group and three in the NC group. The twin newborns had Apgar scores, pHs, and weight within the normal range, as did the singleton newborns. The interventions were the same for all neonates, whether singleton or twin.

### 3.1. Participant Clinical and Demographic Characteristics

The clinical and demographic variables of the participants are described in Table 1. There were no significant differences between the groups. However, it is interesting to note that there were certain trends within the groups. A higher percentage of nulliparous women was found in the NC group compared to the CC group. A higher percentage of women with hypothyroidism was found in the CC group compared to the NC group. However, these differences were not found to be significant. No significant differences were found between the groups in terms of their Robson’s classifications.

The clinical and demographic characteristics of the newborns are listed in Table 2. No statistical differences were found between groups for any of their variables.

The lowest birth weight for newborns delivered by NC section was 1190 g, increasing to 2310 g at 10 days of age. The lowest birth weight for newborns delivered by CC section was 2340 g, which increased to 2375 g at 10 days of age. It is interesting to note that NC neonates gained weight during their first 10 days of life, whereas CC neonates remained below their birth weight at 10 days of life.

### 3.2. Key Findings

NC and CC group comparisons: An early initiation of breastfeeding (spontaneous attachment after birth).

In this context, latching was considered to indicate the early onset of breastfeeding. Infants who received skin-to-skin contact at birth successfully started to breastfeed in 97.6% of cases (Table 3).

NC and CC group comparisons: the continuation of “exclusive” breastfeeding at 10 days and 3/6 months.

This analysis only included the cases where initial exclusive breastfeeding took place (NC *n* = 36 (81.3%)); (CC *n* = 26 (89.6%)). No significant differences between the groups were found regarding exclusive breastfeeding at 10 days after birth. Significant differences between the NC and CC groups were found at 3 and 6 months (Table 4). No significant differences between the groups were found regarding exclusive breastfeeding at 10 days after birth and the administration of artificial supplements, respectively.

Comparison of NC and CC neonates: weight gain at day 10 compared to birth weight.

**Figure 1:**
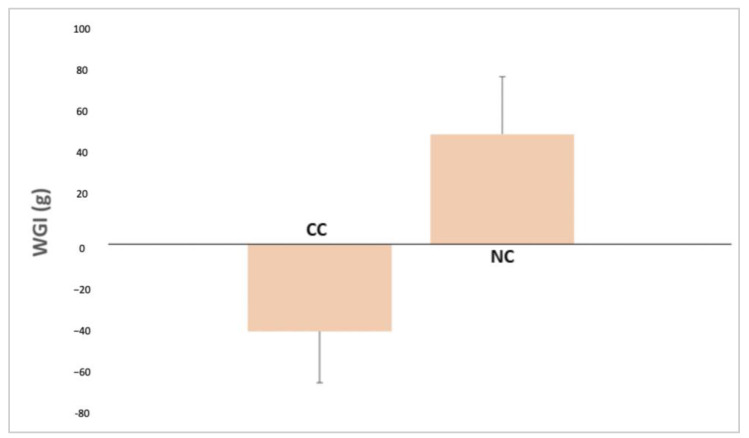
The newborns’ weight at day 10 in the CC and NC group.

The mean weight gain at 10 days was −49.90 g for neonates delivered by NC section and −39.51 g for neonates delivered by CC section. Significant statistical differences were found between the NC and CC groups in terms of neonatal weight gain at 10 days (*p* = 0.015; d Cohen: 0.591) (Figure 1).

Comparison between the NC and CC groups: maternal satisfaction.

Significant differences were found between groups regarding questions, information received, respect from staff, and perceived fear (Table 5).

## 4. Discussion

The present study investigated the influence of the NC approach on maternal/neonatal well-being. The NC approach was found to improve several maternal and neonatal parameters compared to the CC method.

As observed in this study, the separation that occurs between mother and newborn after a cesarean section is considered a barrier to breastfeeding [21,22]. Thus, cesarean delivery is considered one of the major risk factors influencing the early onset of breastfeeding [11,23] due to the physical separation between mother and newborn that occurs after the procedure. In this study, early onset breastfeeding was observed in all participants in the NC group. In this regard, Parry et al. (2013) reported an increase in formula supplementation in newborns delivered by CC section [24]. In our study, a lower percentage of newborns in the NC group received formula supplementation compared to the CC group (22% and 29%, respectively). In addition, skin-to-skin contact is considered to be an effective method for promoting breastfeeding after a cesarean delivery, especially in terms of establishing breastfeeding [25]. In line with previous research [26], this study demonstrated that skin-to-skin contact is critical for maintaining exclusive breastfeeding. The time course analysis of “exclusive” breastfeeding showed higher scores for participants in the NC group at 3 and 6 months. Thus, when newborns who received skin-to-skin contact after a NC section were compared with those who received a CC section, those in the former group breastfed exclusively for longer periods than those in the latter group. Consistent with our results, some other groups have found an interaction between the use of skin-to-skin contact and the duration of breastfeeding [12,27]. Furthermore, these differences would not be justifiable by newborn variables at birth, since, as observed in Table 2, the fetal pH and Apgar scores were normal in all newborns.

The spontaneous latching rate of the newborns was higher in the NC group compared to the CC group. This result can be attributed to the fact that all NC neonates received skin-to-skin contact, and this specific alert stage (window of opportunity) was missed in the CC group. Consistent with this finding, Brown and Jordan, 2013, confirmed that there are greater difficulties in latching in CC neonates due to the methodological factors of the clinical protocol [28].

Another aspect related to breastfeeding is the weight of the newborn. In this regard, neonates born by NC section showed greater weight gain at 10 days compared to neonates born by CC section. These results are consistent with previously published data, showing that the CC protocol results in greater weight loss [29]. Newborn weight gain after NCs may be explained by the fact that this approach provides a context similar to the puerperium of a vaginal birth. The early latching and stimulation of lactational hormones observed after the NC procedure may also explain this difference.

Skin-to-skin contact after a NC section improves maternal satisfaction and reduces anxiety [30,31]. In addition, a reduction in serum reactive oxygen species was found in women who experienced skin-to-skin contact with their newborn [32]. In our study, women in the NC group reported greater satisfaction because they were accompanied by their partner during the surgical and postoperative procedures, were not separated from their baby or their partner, and were able to witness the moment of birth. They reported less anxiety and fear before their cesarean delivery compared to the women who underwent CC sections. In addition, NC mothers felt they received more respect, information, and care from the healthcare professionals in the operating room.

Regarding pain perception during C-sections, participants in the CC group reported higher levels of pain both intraoperatively and postoperatively. This difference in pain perception could be explained by reduced maternal anxiety due to the presence of a birth attendant during the operative and postoperative procedures and the fact that the mother was not separated from her baby or partner during the NC procedure. Our results are consistent with those of other authors who reported reduced pain perception in women who used the skin-to-skin approach immediately after cesarean delivery [33]. However, a controversial study reported no significant differences between the type of cesarean received section and pain perception [17].

The continuous mother–child contact provided by the skin-to-skin approach facilitates the initiation of breastfeeding even in the operating room. Early latching and skin-to-skin contact triggers the release of oxytocin. This hormone is responsible for the subsequent uterine contractions. These early contractions regulate blood loss and prevent postpartum hemorrhage [34]. In this regard, no statistically significant differences in hematological values were found between the groups. This may be explained by the fact that exogenous oxytocin was administered in both types of cesarean section to prevent uterine atony and postpartum hemorrhage. However, a recent study has shown that skin-to-skin contact can increase uterine contractions and improve hemoglobin levels after surgery [35].

## 5. Conclusions

This study highlights the numerous advantages offered by a natural cesarean (NC) compared to a conventional cesarean (CC). Specifically, NC is associated with increased maternal satisfaction with the cesarean process. Mothers reported feeling better informed, experiencing less fear before the intervention, and feeling more respected in the operating room compared to those undergoing conventional cesareans.

Regarding maternal breastfeeding, the NC approach shows greater sustainability, with breastfeeding rates significantly maintained at both 3 and 6 months compared to CCs. Similarly, neonatal weight showed a better recovery and a greater increase at 10 days of life in the NC group.

These findings underscore the significance of incorporating the NC procedure into national health systems, accentuating the potential for optimized and more effective care processes. These insights contribute to the growing body of evidence supporting the adoption of natural cesarean sections, advocating for their widespread implementation across healthcare institutions.

## 6. Limitations

In future studies, it would be advisable to collect data on maternal variables such as mothers’ psychological status, educational level, and external support, among others, as these might influence the continuity of breastfeeding and potentially affect the results obtained.

## Figures and Tables

**Table 1:** Maternal clinical and demographic characteristics.

	NC	CC	*p* Value
Age (mean ± SEM)	35.0 ± 0.82	33.0 ± 0.93	0.606
Nulliparous (%)	51.2	31.0	0.399
Multiparous (%)	48.8	69.0	
Previous breastfeeding experience (%)	48.8	69.0	0.453
Intention to breastfeed after giving birth (%)	84	93.5	0.782
Gestational diabetes (%)	13.9	17.2	0.881
Gestational Hypothyroidism (%)	2.3	6.8	0.330
Robson Classification			0.223
2 group (%) Nulliparous women with a single cephalic pregnancy, ≥37 weeks gestation who had labor induced or delivered by C-section before labor	22	22.6	
3 group (%) Multiparous women without a previous C-section, with a single cephalic pregnancy, >37 weeks gestation, in spontaneous labor	0	3.2	
5 group (%) All multiparous women with at least one previous C-section, with a single cephalic pregnancy, >37 weeks gestation	37.2	51.6	
6 group (%) All nulliparous women with a single breech pregnancy	24.8	9.7	
7 group (%) All multiparous women with a single breech pregnancy including women with previous C-sections	4	6.5	
8 group (%) All women with multiple pregnancies including women with previous C-sections	12	6.5	

Quantitative values are presented as mean ± standard error of the mean, and qualitative values are presented as percentages.

**Table 2:** Newborn clinical and demographic characteristics.

		NC	CC	*p* Value
Multiple gestations		6.81	3.44	0.890
Fetal vein pH		7.35 ± 0.007	7.33 ± 0.007	0.432
Apgar 1′		9.8 ± 0.17	9.7 ± 0.25	0.556
Apgar 10′		9.8 ± 0.03	10.0 ± 0	0.890
Gestational age (%)	Term	98.0	90.3	0.342
	Preterm	2.0	9.5	

Quantitative values are presented as mean ± standard error of the mean, and qualitative values are presented as percentages.

**Table 3:** Frequency and percentage of latching and skin-to-skin contact.

	NC	CC	*p* Value/d Cohen
Latching	41 (97.6%)	0 (0%)	0.000/6.15
Skin-to-skin	48 (96%)	0 (0%)	0.000/8.14

Qualitative values are presented as frequencies and percentages.

**Table 4:** Frequency and percentage of exclusive breastfeeding at 10 days and 3 and 6 months.

	NC	CC	*p* Value/d Cohen
Exclusive breastfeeding at 10 days	27 (67.5%)	14 (50%)	0.208
Exclusive breastfeeding at 3 months	27 (67.5%)	6 (25%)	0.002/0.91
Exclusive breastfeeding at 6 months	12 (50%)	1 (4.5%)	0.001/1.14
Supplementation during the first 10 days of life (mixed breastfeeding)	11 (22%)	9 (29%)	0.16

Qualitative values are presented as frequencies and percentages.

**Table 5:** Descriptive maternal satisfaction.

	NC	CC	*p*-Value/d Cohen
Have you felt adequately informed? *	3.6 ± 0.06	3.0 ± 0.12	0.000/1.179
Were you afraid before your C-section procedure? **	1.8 ± 0.13	1.1 ± 0.22	0.025/0.555
How did you sleep the night before? *	1.6 ± 0.09	1.4 ± 0.14	0.35/1.69
Did you feel well cared for and respected in the operating room? *	3.9 ± 0.02	3.3 ± 0.10	0.000/5.150

* Not at all—0; Slightly—1; Moderately—2; Very—3; Extremely—4; ** Extremely—0; Very—1; Slightly—2; Moderately—3; Not at all—4. Quantitative values are presented as mean ± standard error of the mean.

## Data Availability

The data presented in this study are available on request from the corresponding author.

## References

[1] Rieger-Schemel L. (1989). Protecting, promoting and supporting breastfeeding: The special role of maternity services. J. Hum. Lact..

[2] UNICEF (2018). Protection, Promotion and Support of Breastfeeding in Maternity and Neonatal Facilities: A Review of the Baby Friendly Hospital Initiative.

[3] (2020). The Best Start in Life: Breastfeeding for the Prevention of Noncommunicable Diseases and the Achievement of the Sustainable Development Goals in the WHO European Region.

[4] Ministry of Health and Consumer Affairs (2007). Strategy for Normal Childbirth Care in the National Health System.

[5] (2018). Implementation Guidance: Protecting, Promoting and Supporting Breastfeeding in Facilities Providing Maternity and Newborn Services—The Revised Baby-Friendly Hospital Initiative.

[6] Stivanello E., Rucci P., Carretta E., Pieri G., Fantini M.P. (2013). Risk adjustment for cesarean delivery rates: How many variables do we need? An observational study using administrative databases. BMC Health Serv. Res..

[7] Chu K., Cortier H., Maldonado F., Mashant T., Ford N., Trelles M. (2012). Cesarean Section Rates and Indications in Sub-Saharan Africa: A Multi-Country Study from Medecins sans Frontieres. PLoS ONE.

[8] WHO (1985). Appropriate technology for birth. Lancet.

[9] Betran A.P., Torloni M.R., Zhang J., Ye J., Mikolajczyk R., Deneux-Tharaux C., Oladapo O.T., Souza J.P., Tunçalp Ö., Vogel J.P. (2015). What is the optimal rate of caesarean section at population level? A systematic review of ecologic studies. Reprod. Health.

[10] Crenshaw J.T., Adams E.D., Gilder R.E., Debuty K., Scheffer K.L. (2019). Effects of Skin-to-Skin Care during Cesareans: A Quasiexperimental Feasibility/Pilot Study. Breastfeed. Med..

[11] World Health Organization (2018). WHO Recommendations Intrapartum Care for a Positive Childbirth Experience.

[12] García A., Guerrero E., Hernández M.T., Lagarra C., Martínez-Herrera B., Quintana R. (2017). Working Group of the Clinical Practice Guideline on Breastfeeding: Clinical Practice Guideline on Breastfeeding.

[13] Acuña J., Alba C., Barrio C., López M., Palacios A.P.C. (2010). Care from Birth: Evidence-Based Recommendations and Good Practices.

[14] Smith J., Plaat F., Fisk N.M. (2008). The natural caesarean: A woman-centred technique. BJOG.

[15] Schorn M.N., Moore E., Spetalnick B.M., Morad A. (2015). Implementing Family-Centered Cesarean Birth. J. Midwifery Womens Health.

[16] Armbrust R., Hinkson L., Von Weizsäcker K., Henrich W. (2016). The Charité cesarean birth: A family orientated approach of cesarean section. J. Matern. Fetal Neonatal Med..

[17] Tessier España E., Camaño Gutiérrez I., García Burguillo A., Hernández García J.M., Cotelo R.V., de la Hera Lázaro C., de los Reyes Oliver Pérez M. (2013). Cesárea humanizada. Prog. Obstet. Ginecol..

[18] Hernández S., Basteiro E., Meler E., Cobo T., Figueras F. Protocolo: Cesárea 2020. www.medicinafetalbarcelona.org.

[19] Kelly S., Sprague A., Fell D.B., Murphy P., Aelicks N., Guo Y., Fahey J., Lauzon L., Scott H., Lee L. (2013). Examining Caesarean Section Rates in Canada Using the Robson Classification System. J. Obstet. Gynaecol. Can..

[20] Covas D.M., Alda E., Ventura D.S., Braunstein L.S. (2006). Weight variation during the first month of life in healthy, exclusively breastfed term newborns. Arch. Argent. Pediatr..

[21] Albokhary A.A., James J.P. (2014). Does cesarean section have an impact on the successful initiation of breastfeeding in Saudi Arabia?. Saudi. Med. J..

[22] Badaya N., Jain S., Kumar N. (2018). Time of initiation of breastfeeding in various modes of delivery and to observe the effect of low birth weight and period of gestation on initiation of breastfeeding. Int. J. Contemp. Pediatr..

[23] Esteves T.M.B., Daumas R.P., de Oliveira M.I.C., Andrade C.A.F., Leite I.C. (2014). Factors associated to breastfeeding in the first hour of life: Systematic review. Rev. Saude Publica.

[24] Parry J.E., Ip D.K.M., Chau P.Y.K., Wu K.M., Tarrant M. (2013). Predictors and consequences of in-hospital formula supplementation for healthy breastfeeding newborns. J. Hum. Lact..

[25] Juan J., Zhang X., Wang X., Liu J., Cao Y., Tan L., Gao Y., Qiu Y., Yang H. (2022). Association between Skin-to-Skin Contact Duration after Caesarean Section and Breastfeeding Outcomes. Children.

[26] Zanardo V., Svegliado G., Cavallin F., Giustardi A., Cosmi E., Litta P., Trevisanuto D. (2010). Elective Cesarean Delivery: Does It Have a Negative Effect on Breastfeeding?. Birth.

[27] Spiro A., Neves D.M., Zavala-Soto J.O. (2022). Pro-lactation cesarean section: Immediate skin-to-skin contact and its influence on prolonged breastfeeding. Front. Sociol..

[28] Brown A., Jordan S. (2013). Impact of birth complications on breastfeeding duration: An internet survey. J. Adv. Nurs..

[29] Kelly N.M., Keane J.V., Gallimore R.B., Bick D., Tribe R.M. (2019). Neonatal weight loss and gain patterns in caesarean section born infants: Integrative systematic review. Matern. Child. Nutr..

[30] Salehi A., Fahami F., Beigi M. (2016). The effect of presence of trained husbands beside their wives during childbirth on women’s anxiety. Iran. J. Nurs. Midwifery Res..

[31] Weeks F., Pantoja L., Ortiz J., Foster J., Cavada G., Binfa L. (2017). Labor and Birth Care Satisfaction Associated with Medical Interventions and Accompaniment During Labor Among Chilean Women. J. Midwifery Womens Health.

[32] Yuksel B., Ital I., Balaban O., Kocak E., Seven A., Kucur S.K., Erbakirci M., Keskin N., Assist, Bakirci M. (2016). Immediate breastfeeding and skin-to-skin contact during cesarean section decreases maternal oxidative stress, a prospective randomized case-controlled study. J. Matern. Fetal Neonatal Med..

[33] Phillips R. (2013). The sacred hour: Uninterrupted skin-to-skin contact immediately after birth. Newborn Infant. Nurs. Rev..

[34] Cunningham F. (2010). Obstetricia de Williams.

[35] Pérez-Jiménez J.M., Luque-Oliveros M., Gonzalez-Perez D., Rivera-Sequeiros A., Rodriguez-Blanco C. (2023). Does immediate skin-to-skin contact at caesarean sections promote uterine contraction and recovery of the maternal blood haemoglobin levels? A randomized clinical trial. Nurs. Open.

